# TALEN-Mediated Modification of the Bovine Genome for Large-Scale Production of Human Serum Albumin

**DOI:** 10.1371/journal.pone.0089631

**Published:** 2014-02-21

**Authors:** Shaida Moghaddassi, Will Eyestone, Colin E. Bishop

**Affiliations:** 1 Wake Forest Institute for Regenerative Medicine, Winston-Salem, North Carolina, United States of America; 2 Virginia-Maryland Regional College of Veterinary Medicine, Virginia Tech, Blacksburg, Virginia, United States of America; Institut national de la santé et de la recherche médicale – Institut Cochin, France

## Abstract

As an initial step towards creating genetically modified cattle as a biopharming source of recombinant human serum albumin (rHSA), we report modification of the bovine albumin (bA) locus by transcription activator-like effector nuclease (TALEN)-stimulated homology-directed repair (HDR). Pedigreed bovine fibroblasts were co-transfected with TALENs and an 11.5-kb human serum albumin (HSA) minigene donor construct, designed to simultaneously disrupt and replace bovine serum albumin (BSA) expression with controlled rHSA expression in both the liver and the milk. Targeted integration of the HSA minigene was confirmed in transfected fibroblasts at a frequency of approximately 11% and transgenic bovine embryos were produced from targeted fibroblasts using somatic cell nuclear transfer (SCNT). The research delineated here lays the foundation for the future generation of transgenic rHSA cattle with the potential to provide a large-scale, reliable, and quality-controlled source of rHSA.

## Introduction

There is a vast clinical need for human serum albumin (HSA), with over 500 metric tons used worldwide every year [1], [2]. Currently, HSA is isolated from pooled human blood or plasma, a source that fluctuates unpredictably and can potentially spread human pathogens from donors to recipients [1], [2], [3], [4]. One solution to the problem is the use of transgenic cattle as living bioreactors, enabling the large-scale production of recombinant HSA (rHSA) in a cost-effective manner. Previous bovine transgenics [3] which simply express rHSA in the milk have not proved commercially viable. This is due to the transudation and transcytosis [5] of endogenous bovine serum albumin (BSA), a highly conserved ortholog of HSA, into the milk [6], necessitating an expensive and tedious purification process. Our approach is to humanize the endogenous bovine albumin (bA) gene, replacing it with a dually expressing HSA minigene construct, which will allow for normal expression of rHSA protein in the liver, as well as its exogenous expression in the milk. Such cattle can potentially provide an economically feasible alternative for a safer and more reliable source of therapeutic rHSA.

Until recently, homology-directed repair (HDR) in primary somatic cells has been extremely inefficient [7], [8], [9], [10], [11]. Genome-editing tools, such as zinc-finger nucleases (ZFNs) and transcription activator-like effector nucleases (TALENs), have been shown to significantly improve the efficiency of this process by introducing a site-specific double-strand break (DSB), which greatly stimulates HDR in the presence of a homologous DNA template [10]. The main advantage that TALENs have over ZFNs is that they are designed on a relatively simpler algorithm and are therefore significantly easier and less expensive to manufacture [7], [8], [12], [13]. Using site-specific nuclease technology, genetic modifications, mostly gene disruptions, have been reported in domesticated animals such as sheep, pigs and cattle [7], [9], [11], [14]. We report here the successful use of TALEN-stimulated HDR to introduce an 11.5-kb insert at the bA locus in primary bovine fibroblasts, with an average targeting efficiency of 11%. Furthermore, we show that these targeted cells can be used as nuclear donors in somatic cell nuclear transfer (SCNT) and are capable of directing development to at least the blastocyst stage.

## Results

### Targeting construct design

**Figure 1:**
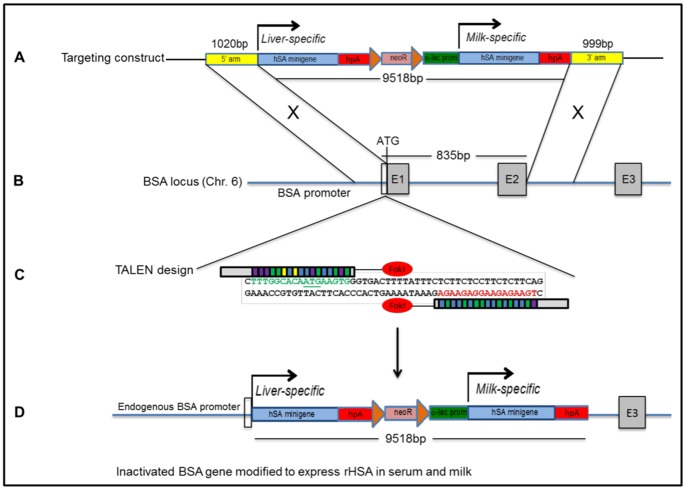
Humanizing the bA locus using TALEN-stimulated HDR. (A) Structure of the 11.5-kb pHSA-neo targeting construct containing two HSA minigenes and 1 kb targeting arms. The bovine α-lactalbumin promoter/regulatory region is 2 kb in size. Diagram is not drawn to scale. (B) Structure of the bA gene on chromosome 6. Targeting arms are homologous to a region immediately upstream of the endogenous 5′translation initiation codon and within intron 2 (C) Binding of the left and right TALENs. TALE repeats are color-coded to represent each of four repeat variable di-residues (RVDs); each RVD recognizes one corresponding DNA base (NI  = A, NG  = T, HD  = C, NN  = G). DSBs are expected to be produced in the 15-bp spacer region in order to stimulate HDR. (D) Proper recombination will result in the deletion of an 835-bp region of DNA, removing bovine exons 1, intron 1, and exon 2, thereby disrupting endogenous bA gene expression and replacing the 5′coding region of the bA gene with two copies of an HSA minigene. HSA1 will be driven by the endogenous bA promoter and will direct rHSA expression to the liver; HSA2 will be expressed in the milk under the α-lactalbumin promoter/regulatory region.

A gene-targeting construct termed pHSA-neo (Figure 1A and Figure S1), which contains two HSA minigenes, was designed to recombine at exon 1 and intron 2 of the bA gene (Figure 1B), resulting in the integration of an 11.5-kb fragment and disruption of endogenous bA gene expression (Figure 1D). The first HSA is a promoterless cDNA that will, after targeted pHSA-neo integration, be driven by the endogenous bA promoter, directing rHSA expression to the liver and ultimately into the blood. The second HSA cDNA will be driven into the milk using a mammary-specific, bovine α-lactalbumin promoter/regulatory region, which has been shown to efficiently direct transgene expression into the milk of mice [15], [16]. In addition, the donor construct contains a centrally located, floxed neomycin-resistance cassette for positive clone selection and an eGFP cassette, located in the vector backbone, which was used as a counter-selection marker for properly recombined cells.

### Evaluation of TALEN efficiency

To stimulate HDR, a TALEN pair (Table S3) was designed to specifically target the bA locus, immediately downstream of the ATG start codon (Figure 1B/C). To evaluate the targeting rate of the TALENs, bovine fibroblasts were transfected with left and right TALEN DNAs and incubated at 30°C for 72 hours, a technique which has been shown to significantly increase the targeting efficiency of site-specific nucleases [17]. A 161-bp DNA fragment spanning the target site was amplified (Figure S2) from extracted genomic DNA (gDNA) and cloned into a pCR™4-TOPO® vector (Invitrogen) for sequencing. Sequencing of clones (N = 48) containing the amplified region showed an indel frequency at the target site of 8.3% (Figure S3). Based on an average transfection efficiency of 70.4% for a similarly sized eGFP-expressing plasmid, we estimate the actual indel frequency to be approximately 11.8%.

### TALEN-stimulated HDR in primary bovine fibroblasts

Two primary bovine fibroblast lines, 3142 (male) and 992 (female), were established from ear skin fibroblasts of pedigreed Holstein calves aged 0.5 and 5 months, respectively. Early passage cells were co-transfected with TALENs and circular pHSA-neo, incubated at 30°C for 3 days [17], and then plated at low-density on 15-cm culture dishes at 37°C under G418 selection. Ring-cloning was performed 10–14 days post-transfection to select for individual, eGFP negative clones. After expansion, individual clones were duplicated for cryopreservation or DNA extraction.

### Analysis of targeted neomycin-resistant fibroblast clones

**Figure 2:**
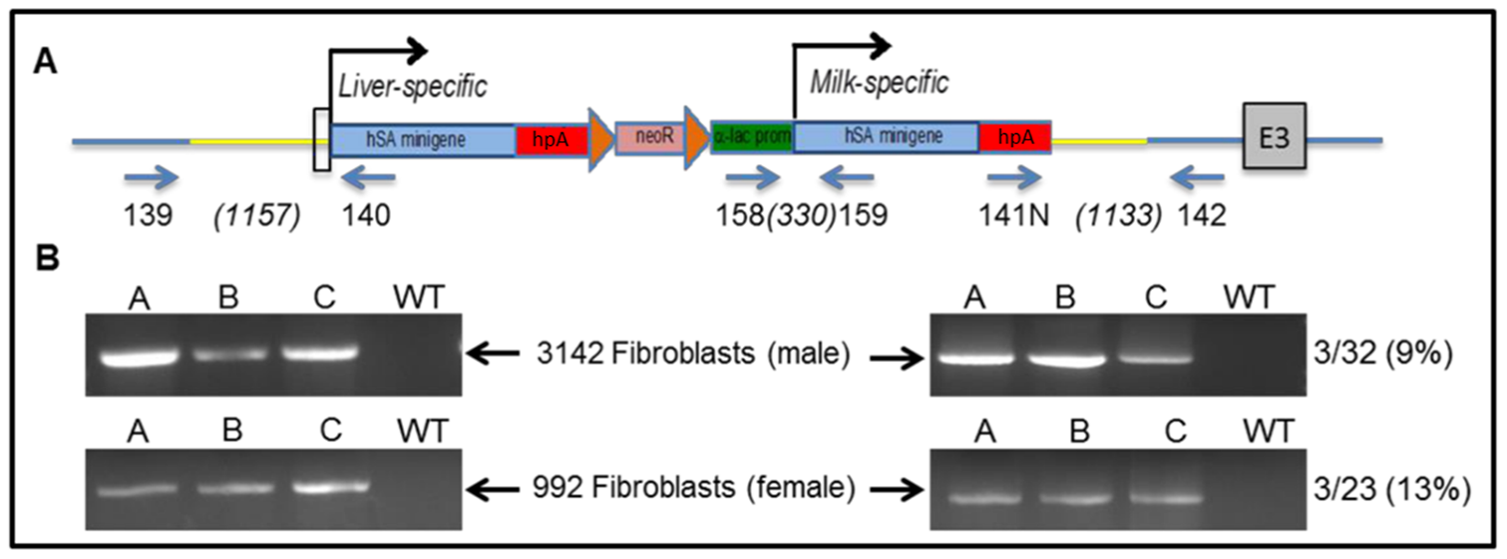
Targeted integration of pHSA-neo at the bA locus. (A) Schematic representation of HDR at the bA gene. Primer 139 is located upstream of the 5′homologous arm and primer 140 is located at the 5′ end of HSA1. Primer 141N is located within the human growth hormone polyadenylation (hpA) sequence of HSA2 and primer 142 is located downstream of the 3′homologous arm. Yellow lines represent the homologous arms of the construct. Figures in brackets indicate the expected product sizes. (B) PCR analysis of HDR at the bA locus for the 5′ (left) and 3′ (right) regions of pHSA-neo. Positive clones derived from the 3142 and 992 cell lines are indicated by A, B, C. Non-transfected (wild-type; WT) DNA from both cell lines was used as a negative control. Targeting efficiency for each cell line is shown at the right.

Correctly targeted clones were identified by PCR (Figure 2) and sequencing analysis using primer sets 139/140 (5′ arm, 1157-bp product) and 141N/142 (3′ arm, 1133-bp product). To distinguish between monoallelic and biallelic targeting, positive clones were analyzed for the presence of the unmodified allele using primers that span the TALEN target site (161 bp). We obtained monoallelic recombination efficiencies of 9.4% (3/32 clones) and 13% (3/23 clones) for the male 3142 and female 992 lines, respectively. Biallelic modifications were not identified in any of the positive clones.

### SCNT

**Figure 3:**
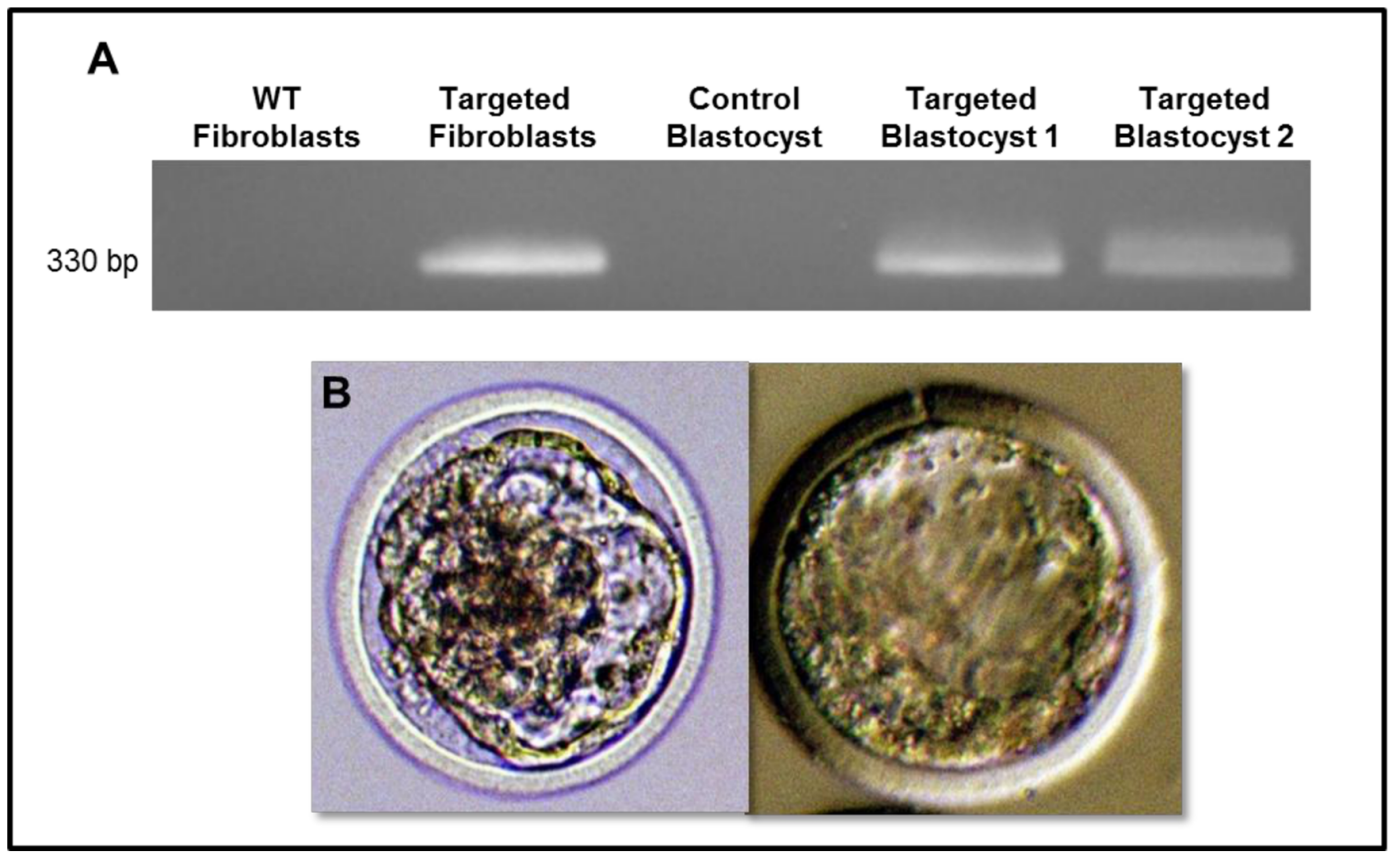
Application of targeted fibroblasts as nuclear donors in SCNT. (A) PCR results demonstrating the presence of the targeted modification in SCNT-generated blastocysts from targeted 3142 fibroblasts. The control blastocyst was generated by SCNT using non-transfected 3142 fibroblasts (WT). One blastocyst was not tested (NT) due to technical issues. (B) Images of the two bovine SCNT-derived blastocysts are shown.

A single clone from the 3142 cell line was used in SCNT to produce transgenic bovine blastocysts, which develop about 7–8 days following nuclear transfer. Out of 51 embryos reconstructed with targeted 3142 fibroblasts, 5 blastocysts were obtained (9.8%) (Table S1). To confirm that the resulting blastocysts contained the same genetic modification as the 3142 nuclear donors, PCR analysis was performed on four of the blastocysts by using the primer set 158/159 to obtain a 330-bp fragment corresponding to the α-lactalbumin promoter/regulatory region and second HSA of the integrated pHSA-neo construct (Figure 3A). Two of the four blastocysts were confirmed to be positive for the pHSA-neo construct. One blastocyst was not analyzed for technical reasons. The two negative blastocysts may have resulted from incomplete SCNT followed by parthenogenic development or the presence of non-targeted cells in the original clones isolated by ring-cloning as previously reported [3].

## Discussion

The massive quantities required to meet the market demand for HSA require an easily scalable system capable of producing high levels of rHSA in a milieu from which it can be inexpensively purified for clinical use in human patients. Production of rHSA has been investigated in several systems [1], [2] including the milk of transgenic dairy cattle [3]. Bovine milk, however, contains significant amounts of BSA that enters the milk from the bloodstream [6]. The close structural similarities of BSA and HSA render rHSA difficult and expensive to purify from bovine milk. Our transgenic strategy addresses this issue in a single step by replacing endogenous BSA with rHSA in the blood, while simultaneously providing a sequence for mammary-specific rHSA expression.

The targeting vector has been designed to maintain potential BSA regulatory sequences, located in the upstream promoter region and/or downstream intronic regions of the endogenous gene, which will remain to optimally regulate expression of the rHSA transgene. Milk from the resulting cows, when bred to homozygosity for the transgene locus, will contain rHSA from both the serum and by mammary synthesis, facilitating simple and inexpensive purification of rHSA from the milk.

Similar work has been achieved in mice, where endogenous mouse serum albumin has been completely replaced with HSA, and serves exclusively as the major circulatory protein in these animals [18]. New Century Pharmaceuticals utilizes these transgenic rHSA mice to evaluate the efficacy, toxicity, immunogenicity, and pharmacokinetics of therapeutic compounds that are intended for clinical use in humans [18]. The successful generation of these healthy, rHSA transgenic mice reinforces the belief that HSA will be capable of supporting life in transgenic cattle. Furthermore, due to the tight homology that exists between HSA and BSA, it is predicted that the two proteins will behave in a comparable manner and will be interchangeable for these purposes.

Before future rHSA cattle can be created using this approach, it is necessary to remove the floxed neomycin-resistance cassette from the modified bovine fibroblasts using a Cre/loxP-mediated system. Due to the fact that the targeted fibroblasts are approaching senescence, it may no longer to be possible to effectively remove the neomycin-resistance cassette by transient expression of Cre recombinase in cell culture. An alternative strategy may be to include a Cre recombinase-expressing plasmid during SCNT, just prior to electrofusion of the oocyte-fibroblast couplet. Furthermore, it would be beneficial to perform a genome-wide evaluation of the modified fibroblasts to test for any unanticipated off-target events, potentially resulting from non-specific TALEN targeting and/or random integration of the pHSA-neo construct.

In conclusion, we have demonstrated that TALEN technology can be used, not only to create simple gene disruptions, but to significantly stimulate HDR in primary fibroblasts, leading to the controlled introduction of large donor constructs. In this instance, we have humanized the bA locus by precisely integrating a large 11.5-kb construct. In addition, we have shown that such modified fibroblasts are capable of producing genetically modified bovine blastocysts using established SCNT technology. Crossbreeding of future genetically modified male and female cattle to generate homozygosity at the modified bA locus can potentially allow for a more reliable and relatively inexpensive source of rHSA, stabilizing worldwide therapeutic supplies and advancing universal improvements in healthcare.

## Materials and Methods

### Derivation of bovine fibroblast cell lines

The primary bovine fibroblast cell lines, 3142 and 992, were established from ear skin biopsies taken aseptically from the auricular margin as a 1 cm ×1 cm ×1 cm triangular biopsy. Harvested biopsies were transported in ice-cold phosphate buffered saline (PBS) to the laboratory for mincing into 1 mm cubes. The harvested tissue was seeded into 100 mm tissue-culture dishes in 10 ml Dulbecco's Modified Eagle Medium (DMEM) supplemented with 10% heat-inactivated fetal bovine serum (FBS) and 10% gentamycin. Explants for all cell lines were cultured for 7–10 days in supplemented DMEM under standard culture conditions of 37°C and 5% CO_2_ in air until cellular outgrowths reached 80% confluency. At this point, cells were harvested by standard trypsinization. Harvested cells were cryopreserved in supplemented DMEM containing 10% dimethyl sulfoxide (DMSO) at passage zero (PO).

### Targeting construct development

The targeting construct, pHSA-neo, was produced in the pSMART low-copy vector (Lucigen Inc.) and propagated using clean genome MDS42drecA *E. coli* competent cells (Scarab Genomics Inc.). The 5′ and 3′ targeting arms, as well as the bovine α-lactalbumin promoter/regulatory region, were generated by PCR from the 3142 bovine fibroblast line. The HSA coding sequences (including their corresponding hpA sites) were obtained from Origene Inc. The PGK/EM7 neomycin-resistance cassette was derived from pL452 (kind gift of Dr. Allen Bradley, Sanger Center UK). The vector was constructed using a combination of standard enzyme restriction/ligation and In-Fusion (Clontech) cloning methods. Finally, the vector was fully sequence-verified (sequence on request) before being used for targeting. The plasmid map for pHSA-neo is depicted in Figure S1.

### Evaluation of TALEN targeting efficiency

TALENs were constructed by Cellectis Inc. according to our design (Table S3). To determine the efficiency of the TALENs to introduce a site-specific DSB, primary bovine fibroblasts (P1) were seeded at an initial concentration of 8×10^4^ per well in a 6-well plate and cultured for 24 hours in DMEM high glucose (Thermo Scientific) containing 10% FBS under standard culture conditions as previously described. Cells were transfected with 1 µg of each TALEN (pCMVTAL_L and pCMVTAL_R) or 2 µg pMAX-GFP (Lonza) using jetPRIME® transfection reagent (Polyplus, France) according to the manufacturer's instructions. Media was replaced on the cells after 4 hours and the cells were transferred to an incubator at 30°C for 72 hours. Following this incubation, gDNA was extracted from TALEN-transfected cells using the Quick-gDNA^TM^ MicroPrep Kit (Zymo Research) according to the manufacturer's instructions. To determine approximate transfection efficiency for the TALENs, cells transfected with pMAX-GFP were evaluated for eGFP expression by FACS analysis at 48 hours post-transfection. Primer set 63/64 was used to amplify a 161-bp region containing the TALEN target site (Figure S2). The generated PCR product was purified using the Nucleospin® Gel and PCR Clean-Up Kit (Clontech), cloned into a pCR™4-TOPO® TA vector (Invitrogen), and subsequently transformed into One Shot® Top10 Competent Cells (Invitrogen). Forty-eight (N = 48) selected clones were subjected to PCR analysis using primers M13F/M13R. The PCR products were purified using the 96 DNA Sequencing Clean-up Kit™ (Zymo Research) according to the manufacturer's instructions and subjected to Sanger sequencing (3130 Applied Biosystems Genetic Analyzer) with primer M13R (Figure S3). Primer sequences are included in Table S2.

### TALEN-mediated HDR in primary bovine fibroblasts

Male (3142) or female (992) primary bovine fibroblasts were seeded in a 15-cm plate at an initial concentration of 2.5×10^5^ and cultured for 48–72 hours under standard culture conditions as previously described. Approximately 2×10^6^ cells from each fibroblast line were transfected using jetPRIME® transfection reagent (Polyplus, France), according to the manufacturer's instructions, with 5 µg of each TALEN vector and 10 µg of pHSA-neo. After 4 hours, media was replaced and the cells were transferred to an incubator at 30°C. After 72 hours incubation at 30°C, the cells were plated at low-density across ten 15-cm plates and returned to standard culture conditions under G418 selection. After 12 days in culture, neomycin-resistant clones were ring-cloned for expansion and analysis. Individual clones were duplicated for DNA extraction (Zymo Research) or cryopreservation.

### Analysis of targeted neomycin-resistant fibroblast clones

Clonal DNA from each cell line was subjected to PCR analysis with primer sets 139/140 (5′end, 1157-bp product) and 141N/142 (3′end, 1133-bp product). Purified PCR products were cloned into pCR™4-TOPO® TA vectors (Invitrogen) and sequenced with primers 139 (5′end) and 142 (3′end) to further confirm HDR at the BSA locus. To determine the occurrence of biallelic targeting, HDR positive clones were analyzed for the presence of a 161-bp region spanning the TALEN target site using primers 63/64 (Figure S2). The amplified region was cloned into a pCR™4-TOPO® TA vector (Invitrogen) for further evaluation by sequencing to elucidate any TALEN-induced indels in the second bA allele.

### SCNT

Immature bovine oocytes were aspirated from slaughterhouse-sourced ovaries and matured *in vivo* for 18 hours. After removing cumulus cells, matured oocytes were stained with Hoescht 33342, incubated in cytochalasin B, and microsurgically enucleated on a Nikon TE-300 inverted microscope equipped with Hoffmann interference contrast optics, Narashige hydraulic manipulators, and UV illumination. Enucleation was confirmed by visualizing the absence of fluorescing metaphase II chromosomes in the oocyte using a brief exposure to UV light. For reconstruction, a single fibroblast was placed microsurgically in the perivitelline space of an enucleated oocyte and subjected to electrofusion using a BTX 2001 cell fusion device (2 pulses of 2 kV/cm, 20 µs each) and mannitol-based fusion medium. Successfully fused embryos were activated by in 5 µM ionomycin for 4 minutes, followed by 4 hours in 2 mM 6-dimethylaminopurine. Activated embryos were cultured for 8 days in modified synthetic oviduct fluid [19] in an atmosphere of 38.5°C, 5% CO_2_, 5% O_2_, and 90% N_2_ for blastocyst development.

### Analysis of SCNT-derived blastocysts

SCNT-derived blastocysts were lysed in 50 mM Tris HCl, 0.5% Triton™ X-100 (Sigma), and 200 µg/ml of proteinase K. The samples were incubated at 55°C for 1 hour to facilitate enzyme activity and heat-inactivated at 94°C for 30 minutes. Samples were stored at −20°C until PCR analysis. The DNA quality of the blastocysts was evaluated using bovine GAPDH primers (9/10). The following 40-cycle PCR was performed with primers 158/159, specific to the α-lactalbumin and second HSA sequences of the targeting construct: 1–2 µl of each experimental blastocyst sample, 1–2 µl of the control blastocyst sample, 100 ng WT 3142 fibroblast gDNA, 50 ng targeted 3142 fibroblast gDNA, and a no-DNA negative control. Results were imaged on a 2% electrophoretic agarose gel. Statistical results for SCNT are shown in Table S1.

## Supporting Information

Figure S1 **Plasmid map of targeting construct, pHSA-neo.**
(PDF)

Figure S2 **Primers used to test for biallelic targeting.** Primer 63 is 10 bp upstream of the endogenous bovine albumin ATG, within exon 1. Primer 64 is 25 bp downstream of exon 1, within the first intron. Expected product size for primer set 63/64 is indicated in parentheses.
(PDF)

Figure S3 **Evaluation of TALEN efficiency.** Sequencing results from analyzed clones (N = 48) revealing TALEN-induced indels at the target site on chromosome 6.
(PDF)

Table S1 SCNT results for targeted and wild-type (WT) 3142 bovine fibroblast donors. One blastocyst from targeted cell line was not tested (NT).
(PDF)

Table S2 **Primer Sequences.**
(PDF)

Table S3 **TALEN sequence information.**
(PDF)
