# Supplementary figures and images for: TALEN-Mediated Modification of the Bovine Genome for Large-Scale Production of Human Serum Albumin
Source: PLoS One. 2014 Feb 21;9(2):e89631. doi: 10.1371/journal.pone.0089631 (PMC3931800; doi:10.1371/journal.pone.0089631)

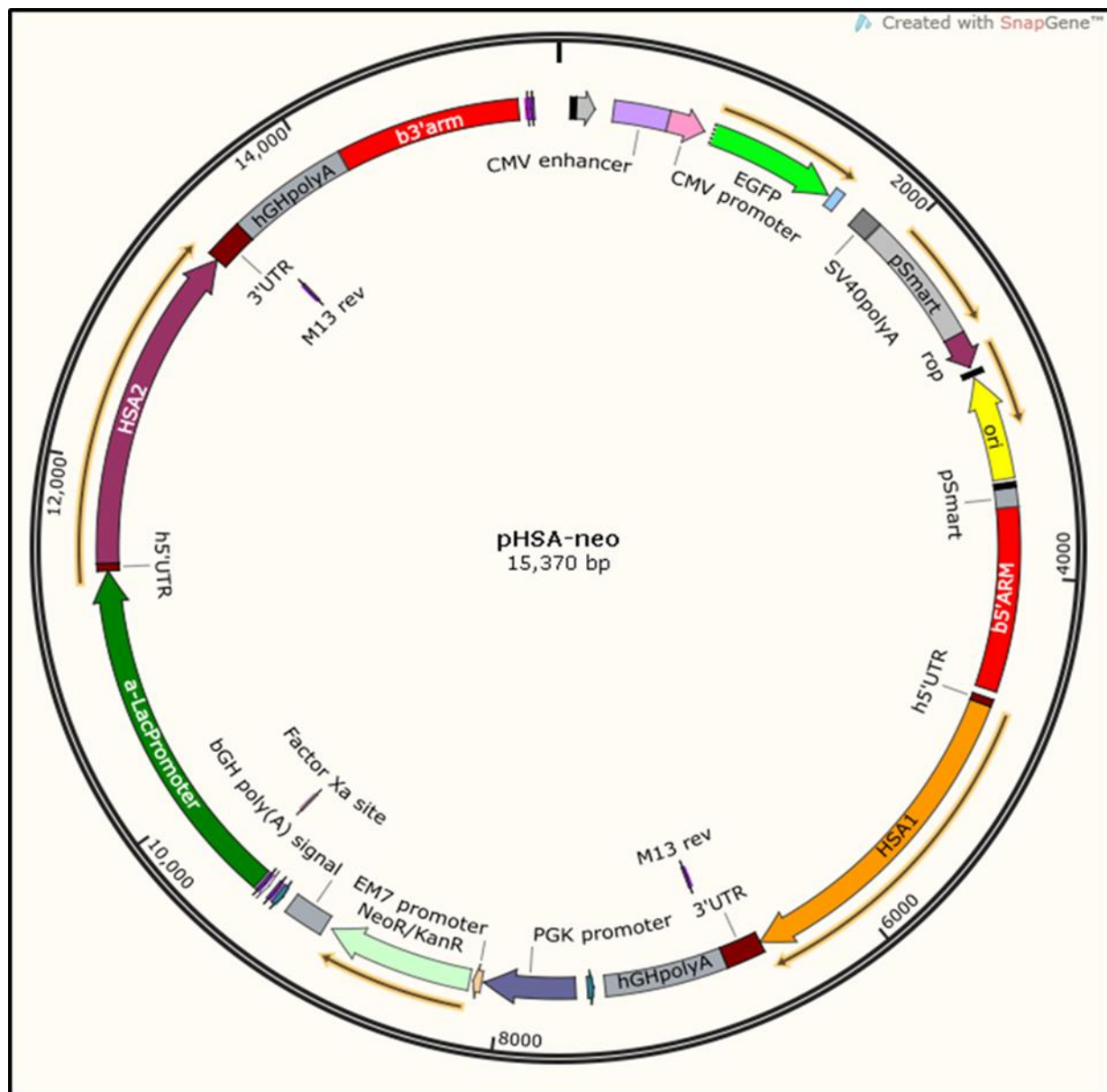

Figure S1. Plasmid map of the targeting construct, pHSA-neo.

Supplement: Figure S1 — Plasmid map of targeting construct, pHSA-neo. (PDF) [file pone.0089631.s001.pdf]
