# Supplementary material for: TALEN-Mediated Modification of the Bovine Genome for Large-Scale Production of Human Serum Albumin
Source: PLoS One. 2014 Feb 21;9(2):e89631. doi: 10.1371/journal.pone.0089631 (PMC3931800; doi:10.1371/journal.pone.0089631)
Supplement: Figure S2 — Primers used to test for biallelic targeting. Primer 63 is 10 bp upstream of the endogenous bovine albumin ATG, within exon 1. Primer 64 is 25 bp downstream of exon 1, within the first intron. Expected product size for primer set 63/64 is indicated in parentheses. (PDF) [file pone.0089631.s002.pdf]

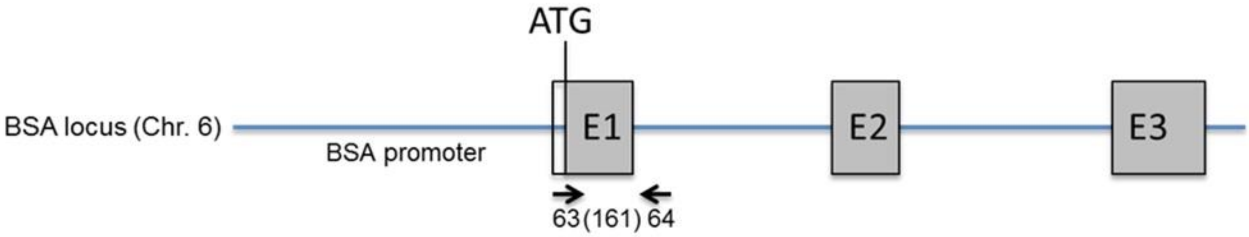

Figure S2. Primers used to test for biallelic targeting. Primer 63 is 10 bp upstream of the endogenous bovine albumin ATG, within exon 1. Primer 64 is 25 bp downstream of exon 1, within the first intron. Primer set 63/64 span the TALEN target site. Expected product size for primer set 63/64 is indicated in parentheses.
