# Supplementary material for: TALEN-Mediated Modification of the Bovine Genome for Large-Scale Production of Human Serum Albumin
Source: PLoS One. 2014 Feb 21;9(2):e89631. doi: 10.1371/journal.pone.0089631 (PMC3931800; doi:10.1371/journal.pone.0089631)
Supplement: Figure S3 — Evaluation of TALEN efficiency. Sequencing results from analyzed clones (N = 48) revealing TALEN-induced indels at the target site on chromosome 6. (PDF) [file pone.0089631.s003.pdf]

WT CTTTGGCACAATGAAGTGGGTGACTTTTATTTCAGAAGAGGAAGAGAAGTG

TALEN-generated indels

CTTTGGCACAATGAAGTGGGTGACTTcTTATTTCTCTTCTCCTTCCTCTTCAG (+1 bp)

CTTTGGCACAATGAAGTGGGTGACTTTTATTTtCTCTTCTTCTCCTTCTCTTCAG (+2 bp)

CTTTGGCACAATGAAGTGGGTGACT-ccTTATTTCTCTTTCTCCTTTCTCTTCAG (-1/+2 bp)

CTTTGGCACAATGAAGTGGGTGAC--TTATTTCTCTTCTCCTTCTCTTCAG (-2 bp)

Figure S3. Evaluation of TALEN efficiency. Sequencing results from analyzed clones (N=48) revealing TALEN-induced indels at the target site on chromosome 6.
