# Supplementary material for: TALEN-Mediated Modification of the Bovine Genome for Large-Scale Production of Human Serum Albumin
Source: PLoS One. 2014 Feb 21;9(2):e89631. doi: 10.1371/journal.pone.0089631 (PMC3931800; doi:10.1371/journal.pone.0089631)
Supplement: Table S1 — SCNT results for targeted and wild-type (WT) 3142 bovine fibroblast donors. One blastocyst from targeted cell line was not tested (NT). (PDF) [file pone.0089631.s004.pdf]

Table S1. SCNT results for targeted and wild-type (WT) 3142 bovine fibroblast donors. One blastocyst from targeted cell line was not tested (NT).

Nuclear Donor	Targeted 3142 Fibroblasts	WT 3142 Fibroblasts
Number fused/number attempted (%)	51/99 (51.5)	35/43 (81.4)
Number developed to blastocyst (%)	5/51 (9.8)	1/35 (2.9)
Number positive for pHSA-neo/number analyzed (%)	2/4 (50)<br>1 NT	0/1 (0)
