# Supplementary material for: TALEN-Mediated Modification of the Bovine Genome for Large-Scale Production of Human Serum Albumin
Source: PLoS One. 2014 Feb 21;9(2):e89631. doi: 10.1371/journal.pone.0089631 (PMC3931800; doi:10.1371/journal.pone.0089631)
Supplement: Table S2 — Primer Sequences. (PDF) [file pone.0089631.s005.pdf]

Table S2. Primer sequences.

Primer Number	Primer Sequence
63	ATCTTTTCTATCAACCCCACAAAAC
64	TCTTGTCTAGGAAAGAAAAGTTGAA
139	TGGGAAGAATAAGCAAGGAACTT
140	TAGAAGATTCTGAAATGGGGTCA
141N	GACATTTGAGTTGCTTGCTTGG
158	CTTACTAGTGTGCCTTCCTTCCCCATGGCG
159	CTTGGATCCCACCTCAGTTTGAATGCATAG
M13F	GTAAAACGACGGCCAG
M13R	CAGGAAACAGCTATGAC
