# Supplementary material for: TALEN-Mediated Modification of the Bovine Genome for Large-Scale Production of Human Serum Albumin
Source: PLoS One. 2014 Feb 21;9(2):e89631. doi: 10.1371/journal.pone.0089631 (PMC3931800; doi:10.1371/journal.pone.0089631)
Supplement: Table S3 — TALEN sequence information. (PDF) [file pone.0089631.s006.pdf]

Table S3. TALEN sequence information.

TALEN	RVD Sequence
Left	NG-NG-NN-NN-HD-NI-HD-NI-NI-NG-NN-NI-NI-NN-NG-NG
Right	NN-NI-NI-NN-NI-NN-NI-NI-NN-NN-NI-NN-NI-NI-NN-NG
